# Magnesium Lithospermate B Protects against Lipopolysaccharide-Induced Bone Loss by Inhibiting RANKL/RANK Pathway

**DOI:** 10.3389/fphar.2018.00064

**Published:** 2018-02-06

**Authors:** Jihai Wang, Xuejian Wu, Yongzhuang Duan

**Affiliations:** Department of Orthopaedics, The First Affiliated Hospital of Zhengzhou University, Zhenghou, China

**Keywords:** lipopolysaccharide, bone loss, magnesium lithospermate B, osteoclast, receptor activator of nuclear factor kappa-B ligand

## Abstract

Lipopolysaccharide (LPS) can induce bone loss by stimulating bone resorption. Natural compounds have great potential for the treatment of osteolytic bone diseases. Magnesium lithospermate B (MLB) plays an important role in protecting against oxidative damage and also has potential anti-inflammatory pharmacological properties. However, its role in LPS-induced bone loss is still unknown. In the present study, we observed the effects of MLB on LPS-induced bone damage and investigated the possible mechanisms. The bone loss models were established by LPS administration in male Sprague–Dawley rats. MLB (200 mg/kg body weight) was given by subcutaneous injection. MicroCT analysis, biomarker assay, histological examination and immunohistochemical staining were performed at the 8th weeks. In addition, RAW264.7 cells were treated with LPS in the presence or absence of MLB. The osteoclast formation, resorption activity and differentiation-related genes [(receptor activator of nuclear factor kappa-B (RANK), Traf6, Fra-1, and c-src)] expression were evaluated. LPS induced bone loss shown as the decrease in bone volume fraction and trabecular number, and increase in trabecular separation. LPS also markedly enhanced the osteoclast formation and resorption activity compared with the control. MLB significantly abolished the LPS-induced bone microstructure damage (*p* < 0.05) and osteoclast formation. MLB also inhibited the increases of serum tartrate-resistant acid phosphatase 5b, RANK ligand (RANKL) and TNF-α level enhanced by LPS (*p* < 0.05). Immunohistochemical staining indicated that MLB attenuated the high expression of RANKL and RANK stimulated by LPS. In addition, MLB significantly abolished the LPS-enhanced osteoclast formation, resorption activity, RANK, Traf6, Fra-1, and c-src expression *in vitro*. Our data demonstrate that MLB can suppress LPS-induced bone loss via inhibiting RANKL/RANK related osteoclast formation.

## Introduction

Lipopolysaccharide(LPS) is a component of the outer membranes of the gram-negative bacteria (Kulp and Kuehn, 2010), which has been identified as the critical pathogenic factor in inflammatory-induced bone resorption (Mori et al., 2013, 2015; Guo et al., 2014). It has been shown that LPS can stimulate bone resorption and suppress bone formation (Bandow et al., 2010). LPS can induce the production of inflammatory factors, such as tumor necrosis factor (TNF)-α and interleukin (IL)-1β, which could promote osteoclast differentiation (Mizutani et al., 2013). The inhibition of LPS-induced osteolysis is critical for the treatment of infective bone diseases (Zhang et al., 2016). Several studies showed that the inhibition of osteoclast formation or activity may be one of treatment strategies (Kim et al., 2014; Zhang et al., 2016).

Recently, many studies show that compounds derived from natural products can prevent bone loss by inhibiting osteoclast formation (Hwang et al., 2013; Zhang et al., 2016; Zhou et al., 2016; Chen et al., 2017). Niu et al. (2017) showed that Nardosinone, a natural compounds from Nardostachys, could inhibited LPS-induced bone loss by inhibiting osteoclast activity. Magnesium lithospermate B (MLB) is an active extract of Salvia miltiorrhiza, which is used to treat coronary heart disease, hepatitis and liver cirrhosis (Zhou et al., 2005). MLB can protect the organs against oxidative damage (Qu et al., 2011) and has anti-inflammatory potential via inhibiting the activation of inflammatory signaling pathways and inflammatory mediators’ expression (Quan et al., 2013; Song et al., 2014). Several studies also showed that MLB has potential for the treatment of ulcer colitis (Jiang et al., 2013, 2016). However, the effect of MLB on LPS-induced bone loss is not clarified.

Receptor activator of nuclear factor kappa-B ligand (RANKL) is a critical cytokine for the formation and activation of osteoclasts (Lacey et al., 1998), which can bind with its receptor RANK and trigger the expression of genes governing osteoclast differentiation (Boyle et al., 2003). Studies also indicated that the inflammatory cytokines induced by LPS could promote the expression of RANKL (Mizutani et al., 2013; Zheng et al., 2014). In the present study, we observe the effects of MLB on LPS-induced bone loss in a rat model and explore the possible molecular mechanisms from the aspect of RANKL/RANK pathway.

## Materials and Methods

### Experimental Design

**FIGURE 1:**
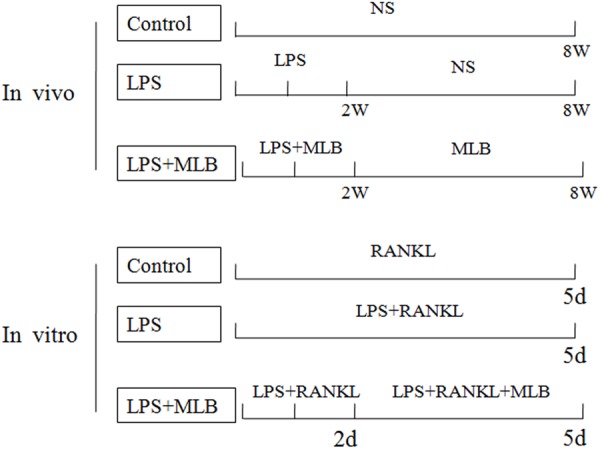
The schematic representation of the experimental design *in vivo* and *in vitro*. *In vivo*, lipopolysaccharide (LPS) was injected intraperitoneally on days 1 and 3 for 2 weeks; Magnesium lithospermate B (MLB) was administrated by subcutaneous injection every other day for 8 weeks. The control group was injected with normal sodium chloride. *In vitro*, RAW264.7 cells were treated with 20 ng/mL RANKL for 5 days, with or without LPS. At the second day, the cells treated with MLB.

Twenty-four specific pathogen free 8-weeks-old Sprague–Dawley male rats weighting about 180 ± 10 g were harvested in the animal facilities under standard conditions (a 12-h light-dark cycle and 21 ± 1°C). After acclimatization to laboratory conditions for one week, they were randomly divided into three groups of eight rats: control group, LPS-treated group, LPS and Magnesium lithospermate B (MLB, purify >85%, Green Vally, Shanghai, China)-treated groups. All animals freely access to water and rat chow. LPS (5 mg/kg) was injected intraperitoneally on days 1 and 3 for 2 weeks; MLB (200 mg/kg body weight) was administrated by subcutaneous injection every other day for 8 weeks. The control group was injected with normal sodium chloride (0.5 ml). The *in vivo* experimental design is shown in **Figure 1**. The *in vivo* experiments were approved by the Institutional Animal Care Committee of the First Affiliated Hospital of Zhengzhou University. All animal experiments comply with the National Institutes of Health guide for the care and use of Laboratory animals (NIH Publications No. 8023, revised 1978).

### MicroCT Analysis

Before sacrifice, MicroCT examinations (Ge eXplore, GE healthcare, United States) was performed in all rats as previous studies described (Chen et al., 2013; Tang et al., 2016). Briefly, rats were placed on the scanning platform after anaesthetizing (7.0% chloral hydrate, 0.5 ml/100 g body weight). The following parameters were used during examinations: tube voltages 80 kV, current 400 μA, a rotation step of 0.5°, field of view 3.2 cm, and 45 μm isotropic resolutions. The left proximal tibia was reconstructed to 3D-rendered images for further analysis. One millimeter distally from the growth plate and extending a further longitudinal distance of 2 mm in the distal direction was selected for quantitative analysis. Cortical bone was avoided during the measurement. Bone volume fractions (BV/TV), trabecular thickness (Tb.Th), trabecular number (Tb.N) and trabecular separation (Tb.Sp) were analyzed by using commercial software (Microview 2.5.0).

### Samples Collection and Preparation

The animals were anesthetized by using chloral hydrate as above. Blood was collected from carotid artery without anticoagulant and centrifuged for serum isolation. The serum was stored at –20°C until analysis. Left tibia was taken off and fixed with 4% polyoxymethylene at 4°C for 3 days after removing the soft tissues. Then, the specimens were decalcified by 10% EDTA at 4°C for 1–2 weeks, dehydrated through a series of ascending ethanol solution (40–100%) and embedded in paraffin. Sections of 5 μm thickness were prepared for Hematoxylin and Eosin (HE) staining or immunochemical staining or tartrate-resistant acid phosphatase (TRAP) staining.

### Histochemistry and Immunohistochemistry

Osteoclast formation in tibia was identified by using TRAP staining as previous study described (Chen et al., 2013; Tang et al., 2016). Briefly, the sections were treated as the following procedures: incubating in oven at 52°C for one hour, dewaxing by using dimethylbenzene, then hydrating through a series of descending ethanol solution (100–40%). TRAP staining was performed following the manufacturer’s instructions (Sigma 387-A, St. Louis, MO, United States). Then, the sections were visualized with a microscope (Nikon 80i, Japan). TRAP positive areas were analyzed by using imaging software (Compix Inc., Irvine, CA, United States).

Receptor activator of nuclear factor kappa-B ligand and RANK expression were evaluated by immunohistochemical staining as previous study described (Chen et al., 2013). Firstly, sections were dewaxed in dimethylbenzene and rehydrated in PBS. Then sections were heated in retrieval solution. Subsequently, the sections were incubated in 1% H_2_O_2_. Next, they were incubated with primary antibodies (Rabbit polyclonal anti-RANKL, 1:150 dilutions; Rabbit polyclonal anti-RANK, 1:100 dilutions, Abcam, Cambridge, MA, United States) overnight at 4°C, following incubation with HRP-conjugated secondary antibodies (1:1000, Abcam, Cambridge, MA, United States) at room temperature. Finally, the antigen-antibody complex was visualized by 3,30-diaminobenzidine (DAB) kit. Then, the sections were viewed with a microscope (Nikon 80i, Japan). RANKL positive area (%) = the area of RANKL positive growth plate/the area of growth plate. RANK positive length = the length of RANK positive bone surface/the length of bone surface.

### Biomarker Determination

Serum tartrate-resistant acid phosphatase 5b (Tracp5b) (Rat TRAP Assay, IDS, United Kingdom), TNF-α and RANKL (R&D systems, Minneapolis, MN, United States) were measured by using EIA methods as the manufacturer’s instructions. For Tracp5b, a standard sample (1.9 U/L) was supplied by manufacturer and the obtained result in our laboratory was 1.8 U/L. The intraassay and interassay were both lower than 5%. For TNF-α and RANKL, the detection limits were both 5.0 pg/ml. The intraassay and interassay were both lower than 5%.

### Cell Cultures

Raw264.7 cells line was obtained from American type culture collection. RAW264.7 cells were cultured in DMEM supplemented with 10% FBS, 1% penicillin/streptomycin, and 20 ng/mL RANKL at 37°C in a humidified atmosphere of 95% air and 5% CO_2_. The RAW264.7 cells were seeded into a 96-well plate at a density of 3.0 × 10^3^ cells/well in the presence of 20 ng/mL RANKL for 5 days, with or without LPS (0.2 μg/ml). At the second day, the cells treated with appropriate concentration of MLB (0, 200, 400 nmol/L). The *in vitro* experimental design is list in **Figure 1**.

### Cell Viability Assay (MTT Assay)

RAW264.7 cells were treated with various concentrations of MLB (20 nmol/L to 200 μmol/L) for 3 days. Then, the medium was removed and cells were added with medium contained 10% MTT solution (Sigma, 0.5 mg/ml in PBS). After a 2-h incubation period at 37°C, the MTT solution was removed and 150 μl dimethyl sulfoxide (Sigma, United States) was added. The optical density (OD) was immediately measured at a wavelength of 570 nm by using microplate reader (Tecan, Austria). The OD values were shown as the mean (six wells for each group).

### Osteoclast Differentiation and TRAP Staining

The cells were fixed with 4% paraformaldehyde for 10 min, and stained for TRAP by using a leukocyte acid phosphatase cytochemistry kit (Sigma–Aldrich, United States) according to the manufacturer’ instructions. The TRAP-positive multinucleated cells containing three or more nuclei were counted as mature osteoclasts by using a microscope (Nikon 80i, Japan).

### Pits Formation

The RAW264.7 cells were seeded on bone wafer in a 24-well plate at a density of 3.0 × 10^3^ cells/well in the presence of 20 ng/mL RANKL for 5 days, with or without LPS (0.2 μg/ml). At the second day, the cells treated with appropriate concentration of MLB (0, 200, 400 nmol/L). Then, cells were wiped-off the bone wafers by using ultrasonic cleaning. Bone wafers were fixed by 2.5% glutaraldehyde for 7 min, stained with 1% toluidine blue for 5 min at room temperature. The pits excavated by osteoclasts were evaluated via a microscope (Nikon 80i, Japan).

### Reverse Transcription Polymerase Chain Reaction (RT-PCR) *in Vivo* and *in Vitro*

Total RNA was extracted from cultured RAW264.7 cells by using Trizol reagent. Then the total RNA was subjected to the synthesis of cDNA by using a Quantscript RT kit (Tiangen Biotech Co., China). The individual cDNA species were amplified in a reaction mixture containing cDNA aliquot, the relevant sense and antisense primers (TRAP: forward 5′-acacagtgatgctgtgtggcaactc-3′, reverse 5′-ccagaggcttccacatatatgatgg-3′; RANK: forward 5′-ccaggacagggctgatgagaa-3′, reverse 5′-tggctgacatacaccacgatga-3′, Traf6: 5′-agcccacgaaagccagaagaa-3′, reverse 5′-cccttatggatttgatgatga-3′, Fra-1: 5′-agagctgcagaagcagaagg-3′, reverse 5′-caagtacgggtcctggagaa-3′; c-src: 5′-ccaggctgaggagtggtact-3′, reverse 5′-cagcttgcggatcttgtagt-3 ′), and SYBR Premix Ex Taq Mix (Takara Bio Inc., Otsu, Japan). Reactions were initiated by incubation at 94°C for 5 min, and PCR (94°C for 5 s, 60°C for 20 s) was performed for 35 cycles.

### Western Blot Analysis

The cells were washed three times with cold PBS buffer, lysed with RIPA Buffer (Beyotime, China) and placed on ice for 30 min. Proteins were separated on SDS-PAGE and subsequently electro transferred to a PVDF membrane (Millipore, United States). The membrane was blocked with 5% BSA in Tris-buffered saline and Tween 20 (10 mM Tris, pH 7.5, 140 mM NaCl, 0.05% Tween-20) for 2 h at room temperature. The membrane was incubated with specific primary antibodies (Rabbit polyclonal anti-RANK, 1:1000 dilution; Rabbit monoclonal anti-Traf6, 1:2000 dilution; Rabbit polyclonal anti-Fra-1, 1:1500 dilution; Mouse monoclonal anti-c-src, 1:1000 dilution, Abcam, Cambridge, MA, United States) followed by horseradish peroxidase-conjugated goat anti-mouse or goat anti-rabbit secondary antibodies. Millipore ECL (Millipore, United States) was used for antibody detection according to manufacturer’s instructions.

### Statistical Analysis

All values were shown as mean ± SD. Statistical significance was determined by Mann–Whitney *U* test. *p* < 0.05 was considered to be statistically significant.

## Results

### MLB Inhibited LPS-Induced Bone Loss

**FIGURE 2:**
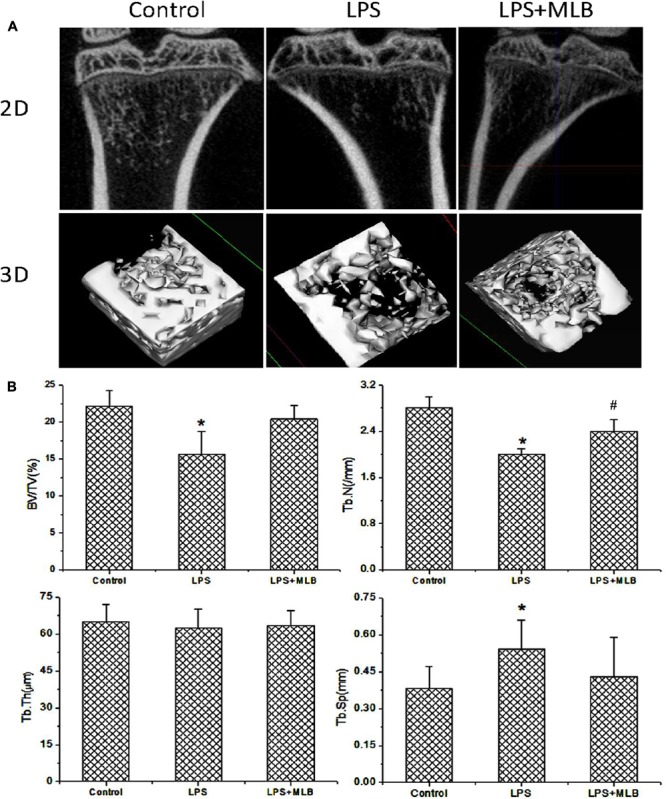
The effects of MLB on LPS induced changes in bone microstructure. **(A)** Two-dimensional (2D) and three-dimensional (3D) reconstructed images derived by micro-computed tomography in control and LPS treated rats with or without MLB. **(B)** Quantitative analysis of bone microstructure parameters based on the reconstructed images derived by micro-computed tomography, including bone volume fraction (BV/TV), trabecular number (Tb.N), trabecular thickness (Tb.Th), and trabecular separation (Tb.Sp). ^∗^*p* < 0.05 or 0.01 vs. other groups; ^#^*p* < 0.05 vs. control.

**FIGURE 3:**
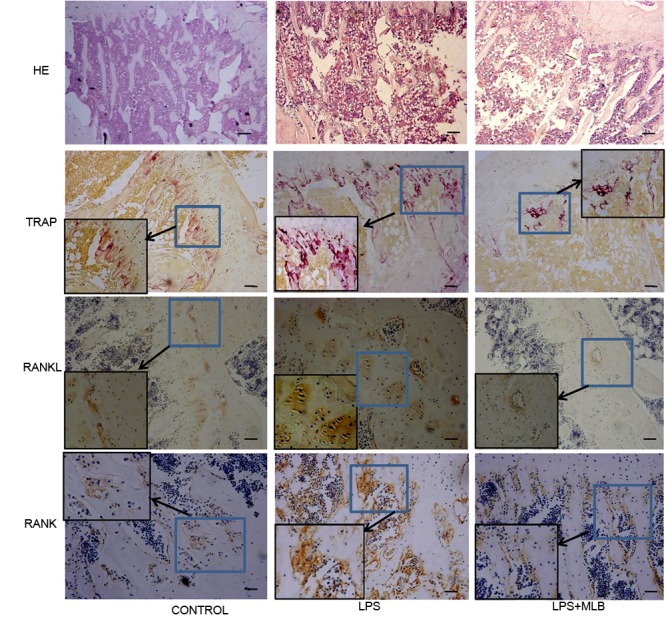
Histologic examinations of tibia using hemotoxylin and eosin staining, Tartrate-resistant acid phosphatase (TRAP) staining and immunochemical staining (RANKL and RANK expression). The immunochemical sections were counterstained with Hematoxylin. LPS: lipopolysaccharide; MLB: magnesium lithospermate B (MLB).

Bone microstructure of tibia was evaluated by microCT (**Figure 2**) and histologic examinations (HE) (**Figure 3**). Qualitative analysis and quantitative data (**Figure 2**) from microCT both showed that LPS induced the reduction in BV/TV, Tb.N, and conjunction points and marked increase in Tb.Sp. On the other hand, MLB treatment significantly dampened the reduction in bone mass as shown by increased BV/TV and Tb.N and decreased Tb.Sp (*p* < 0.05 or 0.01).

### MLB Inhibited Osteoclasts Formation and RANKL/RANK Expression

**FIGURE 4:**
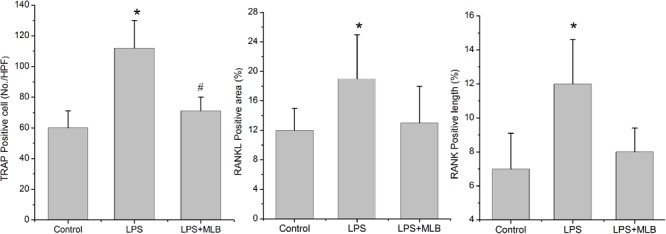
Magnesium lithospermate B inhibited LPS-induced osteoclast formation (TRAP positive cells) and RANKL, RANK expression in bone tissues. ^∗^*p* < 0.05 vs. other groups; ^#^*p* < 0.05 vs. control.

TRAP positive cells were mainly distributed in epiphyseal-metaphyseal region of tibia in the control rats (**Figure 3**). LPS significantly promoted osteoclast formation. The TRAP positive area was higher in LPS-treated rats compared with that of control (*p* < 0.01) (**Figures 3, 4**). The TRAP positive area in rats treated with MLB was significantly decreased compared with rats treated with LPS (**Figures 3, 4**).

Weak RANKL and RANK expressions were observed in control rats. Marked increases in RANKL and RANK expressions were observed in LPS-treated rats (**Figures 3, 4**). However, MLB obviously attenuated the increased expression of RANKL and RANK induced by LPS.

### MLB Decreases Serum Tracp5b, RNAKL, and TNF-α Levels

**FIGURE 5:**
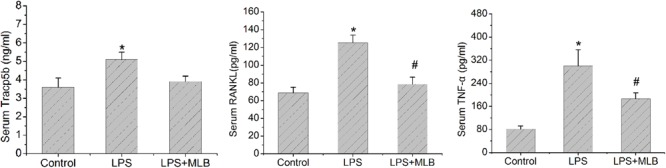
Magnesium lithospermate B inhibited LPS-induced increase in serum Tracp5b, receptor activator of nuclear factor kappa-B ligand (RANKL), and tumor necrosis factor (TNF)-α level. ^∗^*p* < 0.05 or 0.01 vs. other groups; ^#^*p* < 0.05 vs. control.

The levels of serum Tracp5b, RANKL, and TNF-α of rats treated with LPS were significantly higher than that in the control (**Figure 5**), increasing by approximately 30–40% (*p* < 0.05), respectively. MLB significantly reversed the increase of Tracp5b, RANKL, and TNF-α induced by LPS (*p* < 0.05).

### MLB Inhibited RANKL-Induced Osteoclastogenesis

**FIGURE 6:**
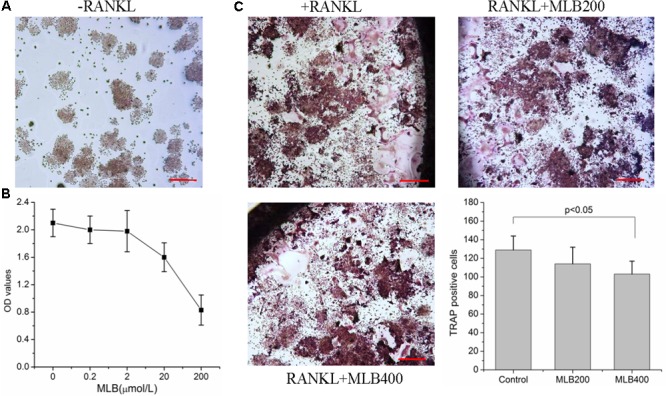
Effects of MLB on viability of RAW264.7 and RANKL-induced osteoclastogenesis. **(A)** RAW264.7 cells could not differentiate into osteoclast without induction of RANKL. **(B)** Cell toxic assay showed that MLB did not affect cell viability at 0.2 to 2 μmol/L. **(C)** The MLB treatment at concentration of 400 nmol/L obviously inhibited osteoclast formation. MLB200: 200 nmol/L; MLB400: 400 noml/L.

RAW264.7 cells could not differentiate into osteoclast without the induction of RANKL (**Figure 6A**). Cell toxic assay (**Figure 6B**) showed that MLB did not affect cell viability at 0.2 to 2 μmol/L. The estimated Lethal Dose 50 was higher than 100 μmol/L. Subsequently, we investigated the effects of MLB on RANKL-induced osteoclastogenesis (**Figure 6C**). The MLB treatment at concentration of 400 nmol/L obviously inhibited osteoclast formation (*p* < 0.05).

### MLB Suppresses LPS Enhanced Osteoclastogenesis *in Vitro*

**FIGURE 7:**
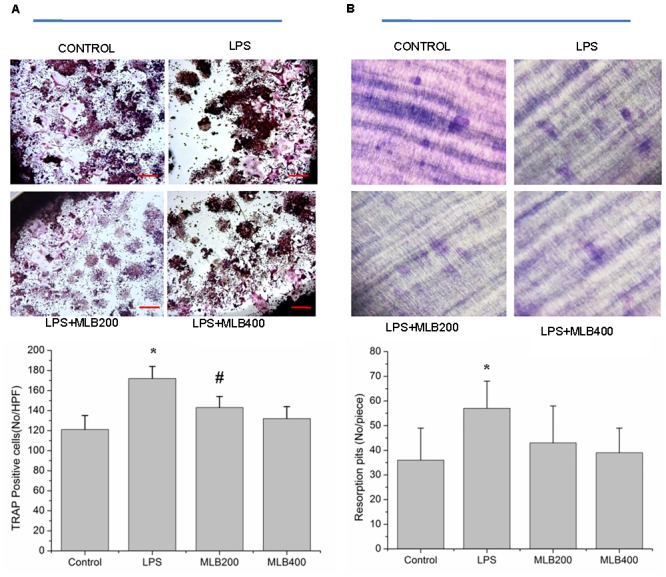
Effects of MLB on osteoclast formation and resorption. MLB inhibited LPS enhanced osteoclast formation (TRAP positive cells) *in vitro* (40×, **A**) and osteoclastic resorption **(B)**. MLB200: 200 nmol/L; MLB400: 400 noml/L. ^∗^*p* < 0.05 or 0.01 vs. other groups; ^#^*p* < 0.05 vs. control.

Lipopolysaccharide enhanced the RANKL induced osteoclast formation (**Figure 7A**) and bone resorption (**Figure 7B**). The MLB at concentration of 200 and 400 nmol/L both inhibited LPS stimulating osteoclastogenesis (*p* < 0.05). MLB treatment at 400 nmol/L also significantly inhibited LPS stimulated bone resorption (*p* < 0.05).

### MLB Inhibited LPS Enhanced RANK, Traf6, Fra1, and c-src Expression

**FIGURE 8:**
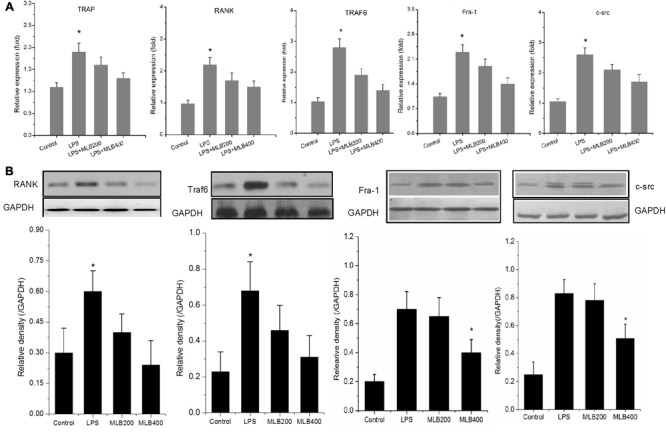
Effects of MLB on LPS-induced changes in RANK signal pathways in RAW264.7 cells. **(A)** MLB inhibited LPS-induced TRAP, RANK, Traf6, Fra-1, and c-src mRNA expression. **(B)** MLB inhibited LPS-induced RANK, Traf6, Fra-1, and c-src protein expression. MLB200: 200 nmol/L; MLB400: 400 noml/L ^∗^ *p* < 0.05 or 0.01 vs. other groups; ^#^*p* < 0.05 vs. control.

To gain further insights into the mechanism by which MLB exerts its inhibitory action on the osteoclast formation and bone resorption, we next examined the influence of MLB on the RANK, Traf6, Fra1, and c-src mRNA (**Figure 8A**) and protein expression *in vitro* (**Figure 8B**). LPS stimulated the mRNA and protein expression of RANK, Traf6, Fra-1, and c-src. The MLB treatment at concentration of 200 and 400 nmol/L both inhibited LPS-stimulated RANK, Traf6, Fra-1, and c-src gene and protein expression, particularly at 400 nmol/L.

## Discussion

Lipopolysaccharide has been identified as a key pathogen of infective osteolytic diseases such as osteomyelitis and septic arthritis (Nason et al., 2009). The inflammatory cytokines induced by LPS can cause bone loss by stimulating osteoclast formation (Zheng et al., 2014; Zhang et al., 2016). Compounds derived from natural products have great potential for inhibiting bone loss by suppressing osteoclast formation (Hwang et al., 2013; Zhang et al., 2016; Zhou et al., 2016; Niu et al., 2017). In the present study, we observed the effects of MLB on LPS-induced bone loss and the possible mechanisms. Our data indicate that MLB protects against bone loss induced by LPS via inhibiting osteoclast formation, which demonstrates that MLB might present an efficient therapy against LPS-induced bone loss. Moreover, we observed that MLB inhibited RANKL, RNAK, and Traf6 expression, which indicates that MLB may suppress the LPS-induced bone loss by inhibiting RANKL/RANK pathway.

Lipopolysaccharide is a stimulator of osteoclastogenesis and bone resorption in infective bone destruction. It can stimulate the production of pro-inflammatory cytokines such as TNF-α, IL-1β, and prostaglandin E2(PGE2), that directly stimulate osteoclast differentiation, and promote excessive bone resorption (Hotokezaka et al., 2007; Mizutani et al., 2013). Osteoclast target-agents have been used to treat LPS-induced bone destruction (Zhang et al., 2016). Currently, many reports showed that the natural compounds derived from Asian herbs can inhibit osteoclast formations (Hwang et al., 2013; Kim et al., 2014; Zhou et al., 2016). Zhou et al. (2016) indicated that Dihydroartemisinin, an anti-malaria drug, could suppress the osteoclast formation and estrogen deficiency-induced bone loss. Several studies showed that emodin, a naturally occurring anthraquinone derivative found in Asian herbal medicines, could suppress osteoclast differentiation and bone resorption (Hwang et al., 2013; Kim et al., 2014; Chen et al., 2017). Recently, several studies also showed that natural compounds, puerarin(Zhang et al., 2016), emodin(Kang et al., 2014; Kim et al., 2014), Sciadopitysin(Cao et al., 2017), Artesunate(Wei et al., 2018), and Nardosinone (Niu et al., 2017) could inhibit LPS-induced bone loss via suppressing the differentiation or activity of osteoclast. In the present study, we demonstrate that MLB also could reverse the excessive osteoclast formation induced by LPS and inhibit the bone loss. It has been shown that MLB plays an important role in protecting against oxidative damage (Qu et al., 2011) and inflammatory mediators’ expression (Song et al., 2014). Our data show that MLB could attenuate the increase of RANKL and TNF-α level induced by LPS, which indicates that the anti-inflammatory pharmacological properties may play important role in MLB effects on LPS-induced bone loss. Previous study also showed that LPS could active NF-κB pathway to induce inflammatory response (Bandow et al., 2010).

Receptor activator of nuclear factor kappa-B ligand is the key cytokine associated with osteoclasts formation and maintaining the survival of mature osteoclast (Lacey et al., 1998; Suda et al., 1999; Boyle et al., 2003). The binding of RANKL to its receptor RANK triggers the activation of signaling pathway related with osteoclast formation and differentiation (Lacey et al., 1998; Boyle et al., 2003). Previous studies showed that LPS could stimulate RANKL expression in osteoblast (Sato et al., 2004) and stromal cells (Ishida et al., 2015), which indicated that LPS may enhance osteoclast formation via stimulating RANKL expression. In the present study, we also show that LPS could up-regulate RANKL expression in osteoblast. In addition, we also observed that RANK expression was significantly increased in LPS treated rats. Moreover, MLB intervention significantly inhibited the high level of RANKL/RANK expression induced by LPS. Therefore, we speculated that MLB may suppress osteoclast formation via inhibiting RANKL/RANK expression. Subsequently, we investigated the effects of LPS and MLB on the four important downstream cytokines of RANKL, RANK, Traf6, Fra-1and c-src *in vitro*. Our data show that LPS could stimulate RANK, Traf6, Fra-1 and c-src expression and MLB markedly reverse the high level of RANK, Traf6, Fra-1, and c-src expression induced by LPS. Previous study also showed that LPS can active NF-κB pathway via upregulating Traf6 (Bandow et al., 2010), which involves in the regulation of osteoclast differentiation and apoptosis. MLB may inhibit the LPS-induced activation of NF-κB pathway. Our data indicate that MLB suppresses osteoclast formation via inhibiting RANKL/RANK pathway. In addition, our data also show that MLB can inhibit RANKL-induced osteoclast formation which indicates that MLB may have potential to treat osteoporosis. Our study is an exploration and further studies using other osteoporosis models, such as ovariectomized osteoporosis are needed.

Our study has several limitations. First, the role of MLB alone on bone was not investigated because we mainly focused on the LPS-induced bone loss. Second, we did not observe the effects of LPS and MLB on osteoblast *in vitro*. We cannot exclude the possibility that LPS and MLB might affect osteoblastic bone formation. Third, the functional evaluations of osteoclast, such as survival and apoptosis *in vitro* were not performed.

## Conclusion

Our data show that MLB can protect against LPS-induced bone loss by inhibiting osteoclast formation. Moreover, our data indicate that MLB may inhibit LPS-induced osteoclast formation by suppressing RANKL/RANK pathway. These data demonstrate that MLB could be explored as a potential therapeutic agent against LPS-induced bone loss.

## Author Contributions

JW designed the experiment and analyzed the data; XW and YD performed the experiments. JW and XW wrote the main manuscript text. JW, XW, and YD explained the results and revised the manuscript. All authors reviewed the manuscript.

## Conflict of Interest Statement

The authors declare that the research was conducted in the absence of any commercial or financial relationships that could be construed as a potential conflict of interest.
